# Antifatigue Effects of *Panax ginseng* C.A. Meyer: A Randomised, Double-Blind, Placebo-Controlled Trial

**DOI:** 10.1371/journal.pone.0061271

**Published:** 2013-04-17

**Authors:** Hyeong-Geug Kim, Jung-Hyo Cho, Sa-Ra Yoo, Jin-Seok Lee, Jong-Min Han, Nam-Hun Lee, Yo-Chan Ahn, Chang-Gue Son

**Affiliations:** 1 Liver and Immunology Research Center, Daejeon Oriental Hospital of Daejeon University, Jung-gu, Daejeon, South Korea; 2 Department of Health Service Management Daejeon University, Dong-gu, Daejeon, South Korea; California Pacific Medicial Center Research Institute, United States of America

## Abstract

The present study investigated the antifatigue effects of *Panax ginseng* C.A. Meyer in 90 subjects (21 men and 69 women) with idiopathic chronic fatigue (ICF) in a randomised, double-blind, placebo-controlled and parallel designed trial. A bespoke 20% ethanol extract of *P. ginseng* (1 g or 2 g day^–1^) or a placebo was administered to each group for 4 weeks, and then fatigue severity was monitored using a self-rating numeric scale (NRS) and a visual analogue scale (VAS) as a primary endpoint. Serum levels of reactive oxygen species (ROS), malondialdehyde (MDA), total glutathione (GSH) contents and glutathione reductase (GSH-Rd) activity were determined. After 4-week, *P. ginseng* administration decreased the total NRS score, but they were not statistically significant compared with placebo (P>0.05). Mental NRS score was significantly improved by *P. ginseng* administrations as 20.4±5.0 to 15.1±6.5 [95% CI 2.3∼8.2] for 1 g and 20.7±6.3 to 13.8±6.2 [95% CI −0.1∼4.2] for 2 g compared with placebo 20.9±4.5 to 18.8±2.9 [95% CI 4.1∼9.9, P<0.01]. Only 2 g *P. ginseng* significantly reduced the VAS score from 7.3±1.3 to 4.4±1.8 [95% CI 0.7∼1.8] compared with the placebo 7.1±1.0 to 5.8±1.3 [95% CI 2.2 ∼3.7, P<0.01]. ROS and MDA levels were lowered by *P. ginseng* compared to placebo. *P. ginseng* 1 g increased GSH concentration and GSH-Rd activity. Our results provide the first evidence of the antifatigue effects of *P. ginseng* in patients with ICF, and we submit that these changes in antioxidant properties contribute in part to its mechanism.

**Trial Registration:**

Clinical Research Information Service (CRIS) KCT0000048

## Introduction

The medically unexplained chronic fatigues, including idiopathic chronic fatigue (ICF) and chronic fatigue syndrome (CFS) are debilitating illnesses due to severe impairment of the quality of life, due to their unknown aetiology and the lack of effective therapeutics [1]. Therefore, many patients with chronic fatigue use alternative and complementary therapies. One study reported that approximately 80% of subjects with chronic fatiguing illness, including ICF and CFS, used alternative and complementary therapies such as massage or herbal supplements in the United States [2].

*Panax ginseng* C.A. Meyer is one of the best known medicinal plants worldwide, and has traditionally been used in Asian countries to maintain homeostasis of the body and to enhance vital energy [3]. About 13.6% of subjects with chronic fatigue ingested ginseng supplements in one Korean study [4]. To date, numerous active compounds (including approximately 40 ginsenosides) have been identified in *P. ginseng* [5]. Also, various pharmacological activities have been identified, such as effects on chemical stress [6] and immune modulation [7] in animal studies, and antitumour activity [8], and effects on glucose metabolism [9] and enhancement of cognitive performance [10] in clinical studies [11].

One group reported anti-mental fatigue effects of *P. ginseng* (G115) by showing improvements in the cognitive performance of healthy volunteers in serial clinical studies [12], [13]. Another group also demonstrated a positive effect of *P. ginseng* (G115) on memory in healthy volunteers [14], [15]. Difficulty in concentration and memory or altered mood is a major symptom of chronic fatigue-associated disorders [16], [17]. The aforementioned studies were conducted in healthy subjects, but these positive results suggest an antifatigue effect of *P. ginseng* in patients with ICF and CFS.

Antifatigue effects of *P. ginseng* have been strongly anticipated from clinical experience and animal-based experiments [18], [19]. Oxidative stress is considered as a main contributor to the pathology of chronic fatigue [20]. Much experimental and clinical evidence supports the antioxidant properties of *P. ginseng* [21], [22]. A randomised controlled trial (RCT) showed an improvement in cancer-related fatigue using another species of ginseng, *Panax quinquefolius* [23]. However, no scientific investigations to date have investigated the antifatigue effects of *P. ginseng*.

Thus, to evaluate the antifatigue effects of *P. ginseng* and its antioxidant activities, we conducted a RCT in 90 subjects with ICF.

## Subjects and Methods

### Ethic Approval

This trial took place in Daejeon Oriental Hospital of Daejeon University after approval of the Institutional Review Board of Daejeon Oriental Medical Centre (authorisation number: DJOMC-51) and was registered on Clinical Research Information Service in Rep. of Korea (KCT0000048). It was conducted in accordance with the Helsinki Declaration of 1975 (as sixth revision in 2008) and the Guidelines for Good Clinical Practice approved from Korea FDA since Aug. 2005 (Approval No. 110). From June, 1^st^, 2010 to January, 31st, 2011, total 90 patients were informed preoperatively about the study via a standardized leaflet and provided written consent for their participation. The protocol for this trial and supporting CONSORT checklist are available as supporting information; see Checklist S1 and Protocol S1.

### Subjects

Adults (age from 20 to 65 years) who had experienced chronic fatigue for longer than 6 months were recruited for the study. A physician and radiologist examined potential candidates and thus excluded those who had any haematological or radiological test abnormalities related to their fatigue. Using the Korean version of the Beck Depression Inventory (BDI) and the Korean translation of the State–Trait Anxiety Inventory (STAI), subjects with a history of psychological disorders or currently experiencing severe depression (BDI score >29) or anxiety (STAI score >70) were excluded [24], [25]. Subjects who worked at night, used alcohol, smoked, took medication or were severely overweight (body mass index >30) were excluded.

**Figure 1:**
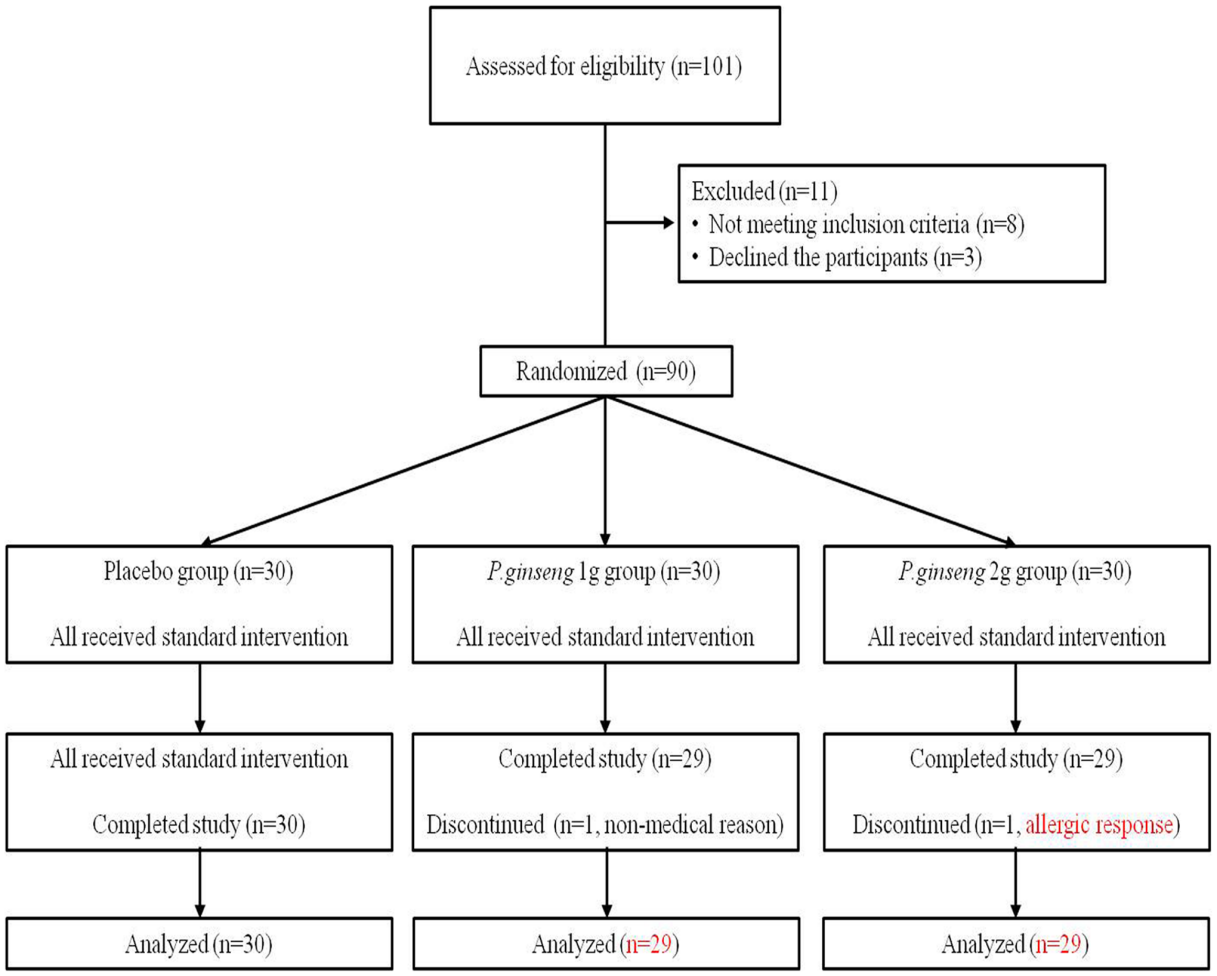
Flow diagram of subject progress through the phases of the RCT.

In this study, the sample size was calculated based on a two-tailed alpha level of 0.05, a power level of 0.90 and 10% drop-out, respectively. The minimal detectable effect size was *r* = 0.53 and the target sample size were 90. Only patients with ICF were selected, while those patients who met the criteria for CFS were excluded. When diagnosing patients with ICF, the criteria of CFS is typically used [26]. CFS is the unexplained chronic fatigue which has concurrently 4 or more of 8 symptoms (post-exertion malaise lasting more than 24 hours; multi-joint pain without swelling or redness; unrefreshing sleep; headaches of a new type, pattern, or severity; significant impairment of short-term memory or concentration; tender cervical or axillary lymph nodes; muscle pain; a sore throat that is frequent or recurring). Patients were diagnosed as ICF when they had only 3 or less of 8 above symptoms. Total 101 patients were recruited for pre-screening, while 90 subjects (21 men and 69 women) were finally enrolled (median age: 39.5 years; range: 20–60 years; mean body weight: 56.7±9.1 kg, mean height 161.5±7.1 cm, Table 1 and Figure 1).

**Table 1:** Physical baseline characters of subjects.

Group	Total	Placebo	*P. ginseng* 1 g	*P. ginseng* 2 g
Subject No. (%)	90 (100)	30 (100)	30 (100)	30 (100)
*Male*	21 (23.3)	6 (20)	9 (30)	6 (20)
*Female*	69 (76.7)	24 (80)	21 (70)	24 (80)
Median age(range)	39.5 (20∼60)	39.5 (24∼60)	39.5 (25∼57)	40.5 (22∼59)
*Male*	44 (25∼59 )	48.5 (41∼58)	43.5 (25∼55)	51.5 (25∼59)
*Female*	37(20∼60)	37.0 (24∼60)	39.0 (31∼57)	38.5 (22∼57)
Body weight (kg)	56.8±9.4	56.9±9.0	59.4±9.9	56.9±9.1
*Male*	68.1±7.3	68.8±8.4	71.1±5.8	64.4±8.5
*Female*	55.3±7.8	55.7±7.1	55.0±8.4	53.5±8.2
Height (cm)	161.5±7.1	160.0±7.1	161.5±6.6	162.5±7.8
*Male*	170.0±5.5	168.5±6.9	170.0±3.0	173.5±7.5
*Female*	160.0±5.7	160.0±5.7	159.0±4.7	161.0±6.4

No significant difference of distribution was observed among groups. Values are means ± SD for body weight and height.

### Study Design

This study was designed as a randomised, double-blind, controlled clinical trial. The randomized allocation was performed with computer-generated block randomization (three, six and nine of block size), performed by a statistical professor from Daejeon University, who was not involved in the data collection and analysis. Ninety of subjects were randomly assigned by a medical staff to three groups as following; a placebo group, 1 g *P. ginseng* group or 2 g *P. ginseng* group.

Each subject ingested four capsules (250 mg each) of *P. ginseng* extract (1 or 2 g total per day, respectively) or the placebo two times a day (09∶00 and 19∶00 h) for 4 weeks. The decision of dosage and duration for administration with *P. ginseng* were based on the previous studies [21], [27]. Assessment of fatigue severity was performed using a numerical self-rating scale (NRS) and a visual analogue scale (VAS) at 0, 2 and 4 weeks. When subjects visited to assess the fatigue severity, they returned the remaining drugs. After the left pill count, staffs offered next pills to each subject.

The serum levels of biomarkers associated with oxidative stress and antioxidants were measured at 0 and 4 weeks. Every subject was subjected to peripheral blood sampling in the Daejeon University Hospital laboratory after an 8-h fast, the morning 1 day before the first and 1 day after the final administration of *P. ginseng* or placebo.

### Preparation and Compositional Analysis of Ginseng Extract and Placebo

A 20% ethanol extract of *P. ginseng* was prepared by the Guryoung Pharmaceutical Company, Ltd. (Cheorwon, South Korea) according to over-the-counter Korean monographs. Briefly, 4-year-old ginseng roots (100 kg) were broken into pieces (100 mesh) and then boiled at 105°C for 6 h. The extract solution was condensed into 20 Brix, and vacuum-freeze-dried. The final extract yield was 13.2% (w/w). The compositional analysis was performed using high-performance liquid chromatography (HPLC), and the results are summarised in Table 2. A placebo was made by mixing 99.36% cornstarch (Daesang Co., Seoul, South Korea), 0.3% *P. ginseng* flavouring (Hanbit Flavor & Fragrance Co., Eumseong-gun, South Korea) and 0.02% caramel colouring (Nam Young Food Co., Busan, South Korea). The 100% *P. ginseng* extract (2 g *P. ginseng*), mixture of 50% *P. ginseng* extract with 50% placebo (1 g *P. ginseng*) and the placebo (control) were put into 250 mg soft capsules.

**Table 2:** Compositional analysis of *P. ginseng* by HPLC.

Ginsenosides	*P. ginseng* Extract (mg/g )	Ginsenosides	*P. ginseng* Extract (mg/g )
Protopanaxadiol		Protopanaxatriol	
Rb3	6.33	Rg1	7.22
Rb1	5.14	Re	2.21
Rb2	3.60	Rg2	0.67
Rc	2.61	Rh1	0.58
Rg3	1.08	–	–
Rd	0.43	*Others* 825.772	
Rh2	0.002	Crude saponins	144.9

*P. ginseng* (1 g) was dissolved in 90% methanol and subjected to HPLC. The column was eluted with solvents A (18% acetonitrile) and B (80% acetonitrile) at a flow rate of 1.6 ml min^–1^. The following solutions were used: 100% A and 0% B changed over 32 min, 80% A and 20% B to 80 min, 0% A and 100% B to 100 min and 100% A and 0% B to 110 min.

### Assessment of Fatigue Severity using *Numerical* Rating *Scale (NRS)* and Visual Analogue Scale (VAS)

The primary endpoint of this study was the change in fatigue severity assessed by measuring NRS and VAS. NRS was used with the Korean-translated Chalder fatigue severity questionnaire [28]. The survey consisted of seven physical health-related questions (1^st^ to 7^th^) and four mental health-related questions (8^th^ to 11^th^) as following: (1) How tired do you feel? (2) How strongly do you currently feel the need to rest? (3) How sleepy or drowsy do you feel? (4) Do you have problems starting things? (5) Are you lacking energy? (6) Do you have less strength in your muscles? (7) Do you feel weak? (8) Do you have difficulty concentrating? (9) Do you have problems thinking clearly? (10) Do you make slips of the tongue when speaking? (11) How is your memory? All subjects scored each item on a 10-point scale (0 = not at all to 9 = unbearably severe fatigue condition). Additionally, patients were asked to indicate their feeling of general fatigue by drawing a vertical line on a 10-cm visual analogue scale (VAS, 0 cm = not at all to 10 cm = unbearably severe fatigue condition).

### Determination of Total Reactive Oxygen Species

The total reactive oxygen species (ROS) level in serum was determined according to Hayashi’s method [29]. Briefly, hydrogen peroxide (H_2_O_2_) was used to generate the standard calibration curve. N,N-Diethyl-para-phenylenediamine (DEPPD) and ferrous sulphate solutions were prepared beforehand. Five microlitres of standard solution or serum was added to 140 µl of 0.1 M sodium acetate buffer (pH 4.8) in 96-well plates and incubated at 37°C for 5 min. One hundred microlitres of DEPPD and ferrous mixture solution were added to each well, and the amount of ROS was determined at 505 nm using a spectrophotometer.

### Determination of Lipid Peroxide as Malondialdehyde

Serum lipid peroxide levels were determined using thiobarbituric acid reactive substances (TBARS), as described by Kamal [30]. TBARS concentration was expressed as µM malondialdehyde (MDA) in serum. Briefly, 250 µl of serum or standard solution was added to 2.5 ml of 20% trichloroacetic acid (TCA) and then mixed with 1 ml of 0.67% thiobarbituric acid (TBA), followed by heating at 100°C for 30 min, cooling on ice and vigorous vortexing with 4 ml *n*-butanol. After centrifugation at 3000×*g* for 20 min, the absorbance of the upper organic layer was measured at 535 nm with a spectrophotometer and compared with a 1,1,3,3-tetraethoxypropane (TEP) standard curve.

### Determination of Total Antioxidant Capacity

Total antioxidant capacity (TAC) was determined according to Kambayashi [31]. Ninety microlitres of 10 mM phosphate-buffered saline (PBS; pH 7.2), 50 µl of 18 µM myogloblin solution and 20 µl of 3 mM 2,2′-azino-bis (3-ethylbenzthiazoline-6-sulphonic acid) diammonium salt (ABTS) solution were mixed with 20 µl of diluted serum samples or various concentrations of gallic acid in a 96-well microplate at 25°C for 3 min. Then, 20 µl of H_2_O_2_ was added to each well and plates incubated for 5 min. The absorbance was measured using a plate reader (Molecular Device Corp., Sunnyvale, CA, USA) at 600 nm. TAC was expressed as gallic acid equivalent antioxidant capacity (GEAC).

### Determination of Superoxide Dismutase and Catalase Activity

Serum superoxide dismutase (SOD) activity was determined using a SOD assay kit (Dojindo Laboratories, Kumamoto, Japan) according to the manufacturer’s protocol. Bovine erythrocyte SOD (Sigma, St. Louis, MO, USA) was used as a standard. Serum catalase activity was determined using the method of Beers and Siezer [32]. Briefly, 100 µl of diluted serum or standard solution was mixed with 2.9 ml of substrate solution (0.0036% [w/w] H_2_O_2_ in 50 mM potassium phosphate), followed by absorbance readings at 240 nm after 5 min.

### Determination of Total Glutathione Contents and Glutathione-peroxidase and Glutathione-reductase Activities

Total GSH content was determined according to a previously described method [33], [34], with slight modifications. Briefly, 50 µl of diluted serum (in PBS 10 mM, pH 7.2) or total GSH standard was combined with 80 µl of DTNB/NADPH mixture (10 µl of 4 mM DTNB and 70 µl of 0.3 mM NADPH) in a 96-well microplate. Next, 20 µl (0.06 U) of GSH-reductase (GSH-Rd) solution was added to each well, and the absorbance at 405 nm was measured using a plate reader (Molecular Devices, Sunnyvale, CA, USA).

GSH-peroxidase (GSH-Px) activity was determined according to the method of Paglia [35]. Briefly, 50 µl of NADPH reagent (5 mM NADPH, 42 mM GSH, 10 units ml^–1^ of GSH-Rd in 1.25 ml of distilled water) was added to 890 µl of GSH-Px buffer (50 mM Tris-HCl, pH 8.0, 0.5 mM EDTA). Then, 50 µl of serum and 10 µl of 30 mM *tert*-butyl hydroperoxide solution were added to the mixture. The final absorbance was measured at 340 nm using a UV-visible spectrophotometer (Varian; Agilent Technologies, Santa Clara, CA, USA). GSH-Rd activity was determined according to the method of Worthington, with slight modifications [36]. Briefly, 150 µl of GSSG with 30 µl of GSH-Rd assay buffer (100 mM potassium phosphate buffer, pH 7.5, with 1 mM EDTA) was added to 30 µl of serum sample and diluted with GSH-Rd dilution buffer (100 mM potassium phosphate buffer, pH 7.5, with 1 mM EDTA and 1 mg ml^–1^ bovine serum albumin). Then, 75 µl of DTNB and 2 mM NADPH were added, and the absorbance at 412 nm was read.

### Statistical Analysis

Based on a power calculation, total 90 subjects were enrolled and randomly allocated into three groups (Placebo, *P. ginseng* 1 g and 2 g) for this trial. Only those subjects who completed the trial were included in the statistical analysis: 30 subjects in the placebo (control), 29 subjects in the 1 g *P. ginseng* and 29 subjects in the 2 g *P. ginseng* groups, respectively. Linear mixed models with compound symmetric covariance [37] were used to compare the combined effects on fatigue symptoms at two time points (2 and 4 weeks). Subgroup analysis was respectively carried out for physical and mental fatigue. Multiple comparisons among groups were performed using the Duncan method when differences among the treatment effects were significant (*P*<0.05). Changes in other biological parameters were examined using one-way ANOVA with PASW Statistics ver. 17 (SPSS, Inc., Chicago, IL, USA).

## Results

### Distribution Patterns

This study was designed as a randomised, double-blind, controlled clinical trial. Ninety subjects were randomly assigned to a placebo group (n = 30) and each dose of *P. ginseng* administration group (1 and 2 g of *P. ginseng,* n = 30 for each group) using a computer generated randomisation schedule. The average of ICF suffering period was similar in all three groups (17.6±12.4 *vs*. 20.8±20.2 *vs*. 19.9±12.5 months in the placebo, 1 g and 2 g *P. ginseng* groups, respectively). The distribution patterns for age, body weight and fatigue severity were normal and even, and there were no statistical significances on the distributions of subjects to each group (Table 1 and 3).

**Table 3:** Analysis of fatigue scores measured by the NRS and VAS.

Measure	Group	Week 0	Week 2	Week 4	95% CI*	*P*-value	Multiple Comparisons
	Placebo	59.3±10.6	52.3±8.4	48.8±7.3	12.9∼25.1		
Total NRS	PG 1 g	58.0±9.1	49.5±11.8	45.5±13.6	6.4∼18.6	0.068	Not done
	PG 2 g	60.8±10.3	45.7±12.3	41.8±13.2	6.4∼14.5		
	Placebo	38.4±8.3	32.4±6.8	30.0±6.7	8.3∼15.8		
PhysicalNRS	PG 1 g	36.9±7.8	31.9±9.7	29.9±9.9	3.0∼11.0	0.119	Not done
	PG 2 g	40.1±7.0	30.5±9.1	28.0±9.0	5.3∼11.4		
	Placebo	20.9±4.5	19.9±3.7	18.8±2.9	4.1∼9.9		Plac. *vs*. PG1: 0.033
MentalNRS	PG 1 g	20.4±5.0	17.0±5.3	15.1±6.5	2.3∼8.2	0.002	Plac. *vs*. PG2: 0.002
	PG 2 g	20.7±6.3	15.3±5.5	13.8±6.2	−0.1∼4.2		PG1. *vs*. PG2: 0.340
	Placebo	7.1±1.0	5.8±1.5	5.8±1.3	2.2∼3.7		Plac. *vs*. PG1: 0.449
VAS	PG 1 g	7.1±1.3	5.6±1.5	5.0±1.4	1.6∼2.7	0.049	Plac. *vs*. PG2: 0.037
	PG 2 g	7.3±1.3	4.9±1.9	4.4±1.8	0.7∼1.8		PG1. *vs*. PG2: 0.405

The subject number of each group was 30, 29 and 29 for placebo, 1 g of *P. ginseng* and 2 g of *P. ginseng* group respectively. Data were expressed as mean ± standard deviation (SD).

* 95% confidence interval (CI) came from the changed values between week 0 and week 4.

### Change in Fatigue Severity as Determined by NRS

The total NRS scores (mean ± standard deviation) were 59.3±10.6, 58.0±9.1 and 60.8±10.3 in the placebo, 1 g *P. ginseng* and 2 g *P. ginseng* groups, respectively, at the initial time point. The total score for the 11 NRS questions gradually decreased in all three groups after 4-week trials, but the difference among the three groups did not reach statistical significance (*P = *0.068, Table 2). In the analysis of subtotal NRS scores for physical (questions 1∼7) and mental health (questions 8∼11), the mental fatigue symptoms, but not physical symptoms, were significantly improved by both 1 g [mean of changed score 5.2±8.1, 95% CI 2.3∼8.2] and 2 g [mean changed score 2.1±5.8, 95% CI −0.1∼4.2] *P. ginseng* compared with the placebo group [mean changed score 6.9±7.9, 95% CI 4.1∼9.9, *P*<0.01] at the final day of trials. No statistical difference between the 1 g and 2 g *P. ginseng* groups was observed (*P*>0.05, Table 3).

### Change in fatigue Severity as Determined by VAS

The initial VAS scores in the placebo and 1 g and 2 g *P. ginseng* groups were 7.0±1.0, 7.1±1.3 and 7.3±1.3, respectively. VAS scores declined in all groups after the 4-week trial; however, the final scores among all three groups were significantly different (*P*<0.05). A multiple comparison analysis showed a significant reduction in VAS score by 2 g *P. ginseng* administration [mean of changed score 1.2±1.5, 95% CI 0.7∼1.8], compared with the placebo [mean of changed score 2.9±2.0, 95% CI 2.2∼3.7, *P*<0.05]. The change in VAS score in the 1 g *P. ginseng* group [mean of changed score 2.2±1.5, 95% CI 1.6∼2.7] was not significant compared with the placebo (Table 3).

### Serum ROS and MDA Levels

The mean initial serum total ROS concentration was about 187∼189 units in each group. After the 4-week trial, administration of 1 g [mean of changed value 35.1±17.9, 95% CI 28.0∼42.1] and 2 g [mean of changed value 42.0±27.9, 95% CI 31.0∼53.1] *P. ginseng* both significantly decreased total serum ROS levels compared with the placebo [mean of changed value 21.9±26.4, 95% CI 11.9∼33.8, *P*<0.05]. On the initial time point, the mean serum MDA levels were 17.5∼18.7 µM among the three groups. After the 4-week trial, the MDA levels were significantly lowered by administration of both 1 g [mean of changed value 4.4±4.7, 95% CI 2.6∼6.1] and 2 g [mean of changed value 5.1±6.0, 95% CI 2.8∼7.3] *P. ginseng*, compared with the placebo [mean of changed value 0.6±7.6, 95% CI −2.2∼3.5, P<0.05 and *P*<0.01, respectively, Table 4].

**Table 4:** Serum levels of oxidative stress and antioxidant system indicators.

Measures	Weeks	Placebo (n = 30)	PG 1 g (n = 29)	PG 2 g (n = 29)	*P*-value	Multiple Comparison
	Week 0	187.1±24.0	187.0±22.1	188.9±17.1		Plac. *vs*. PG1: 0.035
ROS (unit/mL)	Week 4	164.0±16.5	151.0±16.0	146.8±26.9	0.031	Plac. *vs*. PG2: 0.031
	95% CI*	11.9∼33.8	28.0∼42.1	31.0∼53.1		PG1. *vs*. PG2: 0.939
	Week 0	17.5±5.3	18.7±4.5	18.4±6.3		Plac. *vs*. PG1: 0.028
MDA (µM)	Week 4	16.7±5.7	14.3±4.1	13.4±3.7	0.009	Plac. *vs*. PG2: 0.009
	95% CI	−2.2∼3.5	2.6∼6.1	2.8∼7.3		PG1. *vs*. PG2: 0.665
	Week 0	223.7±47.9	237.3±31.4	220.1±31.9		
TAC (µM)	Week 4	239.6±64.9	274.1±88.5	277.0±150.0	0.147	Not done
	95% CI	−44.0∼17.1	−68.8∼−4.9	−113.8∼−5.7		
	Week 0	1.60±0.70	1.03±0.56	1.76±0.52		
SOD (unit/mL)	Week 4	1.25±0.60	0.88±0.34	1.45±0.60	0.784	Not done
	95% CI	0.08∼0.65	−0.06∼0.35	0.08∼0.54		
	Week 0	606.9±167.9	649.6±196.2	572.5±108.4		
Catalase (unit/mL)	Week 4	659.6±127.3	702.8±228.3	601.3±81.9	0.645	Not done
	95% CI	−122.3∼12.5	−144.5∼37.9	−70.5∼13.0		
	Week 0	56.5±9.9	57.4±5.8	60.8±7.9		Plac. *vs*. PG1: 0.043
GSH contents (µM)	Week 4	55.4±7.2	61.1±7.4	61.2±5.1	0.043	Plac. *vs*. PG2: 0.508
	95% CI	−2.5∼5.7	−6.0∼−1.3	−3.8∼2.8		PG1. *vs*. PG2: 0.170
	Week 0	0.10±0.04	0.08±0.04	0.09±0.03		
GSH-Px (unit/mL)	Week 4	0.14±0.04	0.11±0.04	0.12±0.09	0.833	Not done
	95% CI	−0.07∼−0.01	−0.05∼−0.01	−0.07∼0.00		
	Week 0	0.44±0.06	0.37±0.06	0.43±0.04		Plac. *vs*. PG1: 0.021
GSH-Rd (unit/mL)	Week 4	0.41±0.16	0.42±0.05	0.43±0.07	0.021	Plac. *vs*. PG2: 0.595
	95% CI	−0.04∼0.08	−0.07∼−0.02	−0.02∼0.03		PG1. *vs*. PG2: 0.072

Data were expressed as mean ± standard deviation (SD).

* 95% confidence interval (CI) came from the changed values between week 0 and week 4.

### Serum TAC Level and SOD and Catalase Activity

TAC levels, expressed as GEAC, were approximately 220∼237 µM in each group. The serum TAC level was increased in both the 1 g and 2 g *P. ginseng* groups, but the changed values were not significantly different from that of the control group. The serum SOD and catalase activity were about 1.0∼1.8 and 572∼650 units in each group, respectively. The changed values of SOD and catalase activity also showed no noticeable differences among the three groups (Table 4).

### Serum Total GSH Content and GSH-Px and GSH-Rd Activity

Administration of 1 g *P. ginseng* significantly increased serum GSH concentration [from 57.4±5.8 to 61.1±7.4 µM, mean of changed value −3.7±6.2, 95% CI −6.0∼−1.3] compared with the placebo group [56.5±9.9 to 55.4±7.2 µM, mean of changed value 1.7±10.6, 95% CI −2.5∼5.7, *P*<0.05]. In addition, administration of 1 g *P. ginseng* significantly increased serum GSH-Rd activity [0.37±0.06 to 0.42±0.05 units, mean of changed value −0.05±0.07, 95% CI −0.07∼−0.02], compared with the placebo [0.44±0.06 to 0.41±0.06 units, mean of changed value 0.02±0.17, 95% CI −0.04∼0.08], *P*<0.05). However, GSH-Px activities were not changed by *P. ginseng* administration compared to the placebo (Table 4).

### Successful Completion Rates and Adverse Effects

Eighty-eight subjects completed all procedures, including *P. ginseng* administration and blood sampling. One female subject in the 2 g *P. ginseng* group withdrew from the study due to systemic rash and pruritus after 7 days. No one reported other adverse effects. A male subject in the 1 g *P. ginseng* group withdrew for non-health-related reasons. At the end of the trial, 23 subjects in the control group, 17 in the 1 g *P. ginseng* group and 19 in the 2 g *P. ginseng* group speculated that the drug administered was *P. ginseng*.

## Discussion

*P. ginseng* is a popular herbal remedy that has been used in eastern Asian cultures for thousands of years and has been gaining popularity for the treatment of various health-related complaints worldwide [38]. Although ginseng has long been used in clinical settings, the increasing consumption of ginseng products requires more rigorous scientific evaluation to establish appropriate clinical use with regard to efficacy, safety and drug interactions [39]. The present study evaluated the antifatigue effects of *P. ginseng* and its antioxidant activity to explain the mechanism underlying its effects in patients with ICF. Of the two major unexplained chronic fatigue types, ICF generally shows a prevalence 10 times higher than that of CFS, as 4.2% of subjects have ICF and 0.42% of subjects suffer from CFS in the general US population [40].

In order to reduce the fatigue severity of subjects at baseline, the only patients with ICF were involved and patients with CFS were excluded. The primary endpoint of this study was change in fatigue symptoms as measured by total NRS. The fatigue severity in all three groups was comparable, with total NRS scores ranging from 58.0 to 60.8 and VAS scores from 7.0 to 7.3. These scores represent about 60% and 70% of the maximum possible NRS (99 points from 11 questions) and VAS scores, respectively. During the 4-week administration of *P. ginseng* extract or placebo, the total NRS and VAS scores showed a decreasing pattern in all three groups. Although the decreasing tendency of the total NRS score was prominent after administration of 1 g and 2 g *P. ginseng* [95% CI 6.4∼18.6 and 6.4∼14.5, respectively] compared with placebo [95% CI 12.9∼25.1], statistical significance was not reached at the cut off for multiple comparisons (*P* = 0.068). However, the improvement in VAS score was statistically significant after administration of 2 g, but not 1 g, of *P. ginseng* (*P*<0.05).

When we analysed the antifatigue effects of *P. ginseng*, focusing on the two aspects of physical and mental symptoms, *P. ginseng* administration was associated with significant improvement only in the NRS mental score, but not in the NRS physical score. This result is in accordance with results from other clinical studies. Although seven high-quality studies using *P. ginseng* yielded no positive findings with respect to physical outcomes, they did report positive effects on cognitive performance and mood [12], [14], [41]. These results indicate that *P. ginseng* may exert its antifatigue effects through mental improvements. Besides physical fatigue, mental problems, such as difficulties with learning, memory or concentration, are characteristic symptoms of ICF and CFS [42]. In our results, control group also showed the general improvement of fatigue severity. This would result from placebo effects because placebo group speculated that the drug administered was *P. ginseng*. Psychological distress has been regarded as both the main cause and symptom of chronic fatigue [43]; therefore, we excluded subjects with severe depression or anxiety symptoms in their past histories when evaluating the antifatigue effects of *P. ginseng* in patients with ICF in this study.

Although the aetiology of unexplained chronic fatigue is unclear, several recent studies have shown that oxidative stress may be an important contributor to its pathogenesis [40], [44]. Two clinical studies reported increased levels of plasma peroxides and methemoglobin (MetHb) as oxidative stress markers in patients with CFS [20], [45].Therefore, we examined the serum levels of biomakers associated with oxidative stress and antioxidant-associated biomarker profiles before and after 4 weeks of *P. ginseng* administration. ROS are the main oxidative stressors, and MDA is a quantitative marker of lipid peroxidation by ROS [46]. The subjects with ICF in this study had approximately 1.3-fold higher total serum ROS, and fourfold higher serum MDA, levels comparable with healthy subjects in our previous study [21]. Both 1 g and 2 g *P. ginseng* administration for 4 weeks significantly lowered the serum total ROS and MDA concentrations compared with the placebo (Table 4).

Oxidative stressors are normally eliminated by protective mechanisms such as free-radical scavengers, SOD, catalase and the GSH oxidation/reduction system. SOD and catalase catalyse the decomposition of superoxide into H_2_O_2_ and subsequently into water and oxygen [47]. The GSH system plays a central role in multiple defences, particularly via antioxidant-dependent mechanisms, not only against ROS but also against their toxic products [48]. Contrary to our expectations, *P. ginseng* had no effect on serum TAC, SOD or catalase levels, but administration of 1 g *P. ginseng* significantly elevated the total GSH content and GSH-Rd activity compared with the placebo (Table 4). In addition to the GSH system, *P. ginseng* may activate many pathways to exert antioxidant activity related to antifatigue effects because fatigue severity was improved in the 2 g *P. ginseng* group without a corresponding change in total GSH content or GSH-Rd activity.

We previously reported antioxidant effects of *P. ginseng* (same dose and period) in healthy subjects [21]. One clinical study reported an extended activity duration and significant attenuation of serum MDA elevation and altered catalase and SOD activity by administration of *P. ginseng* extract (6 g daily for 8 weeks) to male subjects [49]. Pretreatment with *P. ginseng* polysaccharides reduced the immobility of mice in the forced swimming test and inhibited the alteration of serum MDA level and GSH-Rd activity [19]. Additionally, ginsenoside-enriched American ginseng extract, the second most popular species of ginseng, suppressed the levels of two oxidative stress markers (F2-isoprostane and 8-hydroxy-deoxyguanosine ratios) in healthy volunteers [50]. Notably, the active compounds of Siberian ginseng (*Eleutherococcus senticosus*), which is from the same family but not the same genus as *P. ginseng*, are eleutherosides (not ginsenosides), and no antifatigue effect was observed in a RCT of Siberian ginseng [51]. These results support our findings that the antioxidant properties of *P. ginseng* may be responsible for its antifatigue effects. However, we are unable to determine whether this antioxidant action plays a key role in staving off fatigue, or which active components are responsible for the antifatigue effects of *P. ginseng.* Pretreatment with 20(R)-Rg3, a major active compound of *P. ginseng,* prolonged weight-loaded swimming time without a significant influence on oxidative stress makers [52].

Adverse effects of *P. ginseng* have been reported only rarely, but include headache and sleep and gastrointestinal disorders [39]. One subject in the 2 g *P. ginseng* group complained of body rash and pruritus, and recovered quickly after withdrawing from the trial. This likely resulted from an allergic response to *P. ginseng* and represents an adverse effect. Another subject in the 1 g *P. ginseng* group discontinued intervention for nonmedical reasons. We estimated the compliance to be over 90% because of the preference of Koreans for *P*. *ginseng*; despite this, two subjects withdrew. Above two subjects were dropped before the first time point of measurement at 2-week. For this reason, we analysed the results per protocol (PP), instead of intention to treat (ITT).

To our knowledge, this study is the first RCT to provide systematic evidence of the antifatigue properties of *P. ginseng* in subjects with ICF. Taken together, these data lead us to conclude that *P. ginseng* can be used to combat chronic fatigue and that the mechanism underlying this effect may be related to its antioxidant properties.

## Supporting Information

Checklist S1 **CONSORT checklist.**
(ZIP)

Protocol S1 **Trial protocol.**
(ZIP)
