# Supplementary material for: Antifatigue Effects of Panax ginseng C.A. Meyer: A Randomised, Double-Blind, Placebo-Controlled Trial
Source: PLoS One. 2013 Apr 17;8(4):e61271. doi: 10.1371/journal.pone.0061271 (PMC3629193; doi:10.1371/journal.pone.0061271)
Supplement: Protocol S1 — Trial protocol. (ZIP) [file pone.0061271.s002.zip › Trial Protocol.doc]

**Clinical Trial Protocol**

**(Translation from Korean to English, Summary)**

**PRINCIPAL INVESTIGATOR**

Chang-Gue Son, Professor, OMD, PhD.

**PROTOCOL TITLE**

A Clinical Study for the Anti-fatigue Effect of Korean Ginseng Water Extract

**FUNDING**

National Research Foundation from the Republic of Korea’s Ministry of Education, Science and Technology.

**VERSION DATE**

March 1^st^ 2010

**SPECIFIC AIMS**

This study aims to determine whether Korean Ginseng can improve the fatigue severity in subjects with idiopathic chronic fatigue. In addition, this trial aims to investigate the underlying mechanism responsible for the anti-fatigue effects of Korean Ginseng, especially regarding antioxidant properties of Korean Ginseng.

**BACKGROUNDS AND SIGNIFICANCE**

Korean ginseng, Panax ginseng C.A. Meyer, is one of the most renowned herbal plants worldwide and has been used for thousands of years to maintain homeostasis of the body and enhance vital energy, particularly in Asian countries. To date, numerous studies have demonstrated the pharmaceutical effects of P. ginseng on physical-, chemical-, and biological-stress, systemic immune function, glucose metabolism, sexual function, and mental capacity.

**On the other hand, the medically unexplained chronic fatigue, including idiopathic chronic fatigue (ICF) and chronic fatigue syndrome (CFS) are debilitating illnesses due to severe impairment of the quality of life, compounded by their unknown** aetiology **and the lack of effective therapeutics.** About 13.6% of subjects with chronic fatigue ingest ginseng supplements in a Korean study. Several groups have evidenced anti-mental fatigue effects of P. ginseng (G115) by showing the improvements of cognitive performance and memory activity in healthy volunteers from serial clinical studies.

Oxidative stress is considered as a main contributor to the pathology of chronic fatigue. Much experimental and clinical evidence supports the antioxidant properties of **P. ginseng**. A randomised double-blind trial (RCT) showed the improvement of cancer-related fatigue using another species of ginseng, Panax quinquefolius.

However, there have been no scientific investigations into the anti-fatigue effects of P. ginseng to date. In order to evaluate the anti-fatigue effects of P. ginseng, and its antioxidant activities, we conducted a RCT in 90 participants with ICF.

**RESEARCH DESIGN AND METHORD**

**Study design:** Randomized, placebo-controlled, double-blinded, Tree-arms study

**Subjects**: Adults who had experienced chronic fatigue for longer than 6 months will be recruited for the study. A physician and radiologist will examine the potential candidates and thus will exclude subjects who have any haematological or radiological test abnormalities related to their fatigue. Only ICF patients will be selected, while those patients who meet the criteria for CFS will be excluded. Using the Korean version of the Beck Depression Inventory (BDI) and the Korean translation of the State–Trait Anxiety Inventory (STAI), subjects with a history of psychological disorders or currently experiencing severe depression or anxiety will be excluded.

**Inclusion and exclusion criteria:** This study includes adults (aged 20 to 65 years) who had experienced chronic fatigue for longer than 6 months.

If subjects meet the criteria for CFS will be excluded.

Four or more of the following symptoms:

- Self-reported impairment of short-term memory or concentration
- Sore throat
- Tender lymph nodes
- Muscle pain
- Multi-joint pain without swelling or redness
- Headaches of a new type, pattern, or severity
- Un-refreshing and/or interrupted sleep
- Post-exertion malaise (a feeling of general discomfort or uneasiness) lasting more than 24 hours

Subjects who work at night, used alcohol, smoked, took medication, or overweight (body mass index >30) will be excluded. Subjects with a history of psychological disorders or currently experiencing severe depression (BDI score >29) or anxiety (STAI score >70) will be excluded.

**Number of participants and randomization:** Ninety subjects will be included finally, and they will be randomized into three groups, placebo, low or high dose P. ginseng group, using a computer-generated randomisation schedule.

**Treatment and period:** Each subject ingestS four capsules (250 mg each) of *P. ginseng* extract (1 or 2 g total per day, respectively) or the placebo two times a day (9:00 AM and 7:00 PM) for 4 weeks.

**Assessment of fatigue severity (Primary measurement):** The primary endpoint of this study was the change in fatigue severity by measuring a numerical self-rating scale (NRS) and a visual analogue scale (VAS) at 0, 2, and 4 weeks, using Korean-translated Chalder fatigue severity questionnaire. All subjects scored each item on a 10-point scale (0 = not at all to 9 = unbearably severe fatigue condition). The survey consisted of seven physical-health-related questions and four mental-health-related questions as follows:

(1) How tired do you feel?

(2) How strongly do you currently feel the need to rest?

(3) How sleepy or drowsy do you feel?

(4) Do you have problems starting things?

(5) Are you lacking energy?

(6) Do you have less strength in your muscles?

(7) Do you feel weak?

(8) Do you have difficulty concentrating?

(9) Do you have problems thinking clearly?

(10) Do you make slips of the tongue when speaking?

(11) How is your memory?

For VAS score, patients will be asked to indicate their feeling of general fatigue by drawing a vertical line on a 10-cm visual analogue scale (0 cm = not at all to 10 cm = unbearably severe fatigue condition).

**Measure of serum oxidative stress/antioxidants biomarkers:** The serum levels of biomarkers associated with oxidative stress and antioxidants are measured at 0 and 4 weeks. Every participant was subjected to peripheral blood sample in laboratory of Daejeon University Hospital under 8-hour fasts in the morning of one day before 1^st^ administration and one day after last administration of P. ginseng. Biomarkers include total reactive oxygen species, malondialdehyde, total antioxidant capacity, superoxide dismutase, catalase activity, total glutathione content, glutathione peroxidise, and glutathione reductase activities.

**Statistical analysis:** Linear mixed models with compound symmetric covariance to compare comprehensively the combined effects on fatigue symptoms at the two time points (2- and 4-weeks). Additionally, multiple comparisons using the Duncan method will be adapted.

**GINSENG EXTRACT AND PLACEBO**

A 20% ethanol extract of P. ginseng, by the Guryoung Pharmaceutical Company, Ltd. (Cheorwon, South Korea) according to over-the-counter Korean monographs. The final extract yield was 13.2% (w/w). A placebo was made by mixing 99.36% corn starch (Daesang Co., South Korea), 0.3% P. ginseng flavouring (Hanbit Flavor & Fragrance Co., South Korea), and 0.02% caramel colouring (Nam Young Food Co., South Korea).

**MINIMIZATION OF RISK**

Korean ginseng is the most common drug which Korean has used for thousand years without any specific adverse effects. Nevertheless, this study will pay attention the risk of any adverse effects.

**RECRUITMENT PROCEDURES**

We will utilize advertisements to recruit subjects with ICF. All advertisements will be submitted to the IRB for approval before use. Those who are interested in joining a participants group will visit Liver & Immunology Research Center for a scheduled screening visit during which informed consent will be obtained by the study physician.

**CONSENT PROCEDURES**

The study will be discussed with ICF patients in the Liver & Immunology Research Center by their clinic physicians during their regular visits. If the patients express interest to their physicians, a study coordinator will meet with them and describe the study. Patients who are interested will be provided with the informed consent to take home. Those who are interested will return to the Liver & Immunology Research Center for a scheduled screening visit during which informed consent will be obtained by the study physician.

**CONTACT INFORMATION:**

Liver & Immunology Research Center, Daejeon Oriental Hospital of Daejeon University
22-5 Daeheung-dong, Jung-gu, Daejeon, Korea 301-724

Tel: 82-42-229-6807/ 42-42-254-3403(fax)
